# Intralymphatic Glutamic Acid Decarboxylase-Alum Administration Induced Th2-Like-Specific Immunomodulation in Responder Patients: A Pilot Clinical Trial in Type 1 Diabetes

**DOI:** 10.1155/2018/9391845

**Published:** 2018-05-24

**Authors:** Beatriz Tavira, Hugo Barcenilla, Jeannette Wahlberg, Peter Achenbach, Johnny Ludvigsson, Rosaura Casas

**Affiliations:** ^1^Division of Pediatrics, Department of Clinical and Experimental Medicine, Faculty of Medicine and Health Sciences, Linköping University, Linköping, Sweden; ^2^Department of Endocrinology and Department of Medical and Health Sciences and Department of Clinical and Experimental Medicine, Linköping University, Linköping, Sweden; ^3^Institute of Diabetes Research, Helmholtz Zentrum München, and Forschergruppe Diabetes, Klinikum Rechts der Isar, Technische Universität München, München, Germany; ^4^Crown Princess Victoria Children's Hospital, Region Östergötland, Linköping, Sweden

## Abstract

GAD-alum given into lymph nodes to type 1 diabetes patients participating in an open-label pilot trial resulted in preservation of C-peptide similar to promising results from other trials. Here, we compared the immunomodulatory effect of giving GAD-alum directly into lymph nodes versus that induced by subcutaneous administration. Samples from T1D patients (*n* = 6) who received 4 *μ*g GAD-alum into lymph nodes (LNs), followed by two booster injections one month apart, and from patients (*n* = 6) who received two subcutaneous injections (SC) (20 *μ*g) given one month apart were compared. GADA, IA-2A, GADA subclasses, IgE, GAD_65_-induced cytokines, PBMC proliferation, and T cell markers were analyzed. Lower doses of GAD-alum into LN induced higher GADA levels than SC injections and reduced proliferation and IgG1 GADA subclass, while enhancing IgG2, IgG3, and IgG4. The cytokine profile was dominated by the Th2-associated cytokine IL-13, and GAD_65_ stimulation induced activated CD4 T cells. Patients responding clinically best account for most of the immunological changes. In contrast, SC treatment resulted in predominant IgG1, predominant IFN-*γ*, higher proliferation, and activated CD4 and CD8 cells. Patients from the LN group with best metabolic outcome seemed to have common immune correlates related to the treatment. This trial is registered with DIAGNODE (NCT02352974, clinicaltrials.gov) and DIABGAD (NCT01785108, clinicaltrials.gov).

## 1. Introduction

Type 1 diabetes (T1D) treatment consists of lifelong administration of insulin, a replacement therapy which does not satisfactorily prevent serious complications. Immune intervention trials in recent-onset T1D patients to delay or halt disease progression have shown no or limited efficacy [1–7], highlighting the complexity of translation from animal models to human T1D. Immunomodulation with autoantigens could potentially constitute the most specific and safe treatment for T1D. Subcutaneous administration of glutamic acid decarboxylase (GAD)_65_ formulated with aluminum hydroxide (GAD-alum) showed efficacy in preserving residual insulin secretion in children and adolescents with recent-onset T1D [8], but subsequent phase II [9] and phase III [10] trials failed to reach their primary outcomes. However, significant efficacy was shown in prespecified subgroups in the phase III study [10], and we have recently shown that close administration of influenza vaccine might have influenced the study outcome [11]. Thus, it is plausible that treatment with GAD-alum is beneficial for some patients but has not been sufficiently effective [12].

Immunotherapy with allergens has been extensively used as treatment for allergic rhinitis and asthma [13, 14]. The most common forms of allergen immunotherapy have been through subcutaneous or sublingual allergen administration [15, 16], but even though they are quite safe and effective, recent studies have focused on further optimization of allergen vaccines to improve clinical efficacy. Intralymphatic administration of low doses of allergens has shown promising result, providing improved allergen exposure and triggering prompt immune changes [17–20]. The mechanism for this form of treatment remains unclear, although immune modulation of the immune responses and induction of tolerance has been suggested [21]. It is known that intralymphatic administration of antigens can maximize immunogenicity and hence efficacy and has shown to improve the efficacy of several vaccines [22, 23]. Thus, to render the presentation of GAD_65_ antigen to T cells in the lymph nodes more efficient than previously described [9, 10], GAD-alum was administrated into lymph nodes to six patients participating in an open-label clinical trial. Results from these patients showed that preservation of C-peptide appeared to be similar to promising results observed in patients from other immune intervention trials [24]. Here, we studied the immunomodulatory effect of giving low doses of GAD-alum directly into lymph glands versus that induced by subcutaneous administration of larger doses.

## 2. Methods

### 2.1. Participants

**Figure 1:**
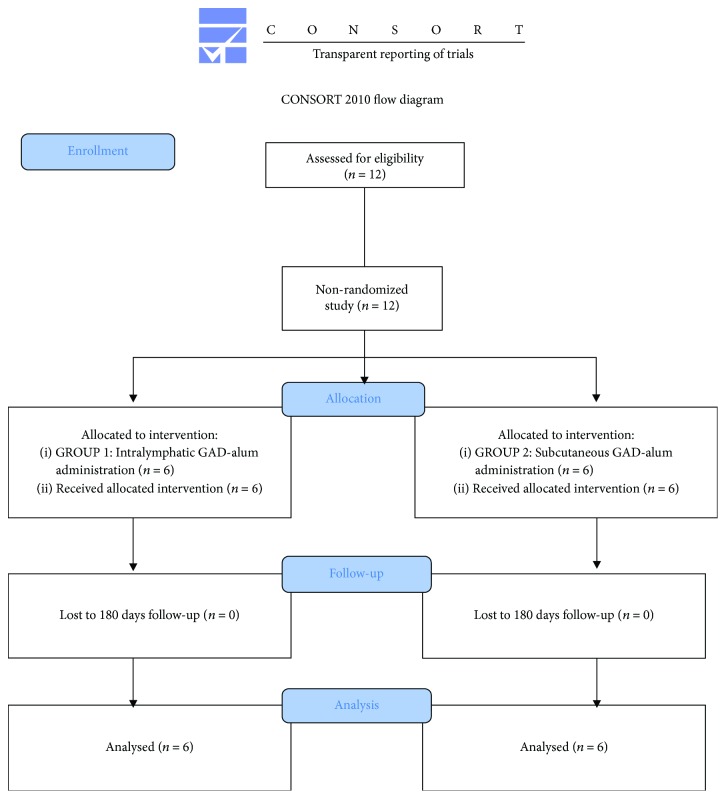
CONSORT flow diagram. A total of 12 patients were recruited in two different clinical studies (*n* = 6 each study) using different doses and administration of GAD-alum. None of the patients were excluded during these trials' follow-up.

A group of recent-onset T1D patients (*n* = 6, <6 months from diagnosis) participating in a pilot trial (NCT02352974, clinicaltrials.gov) received a primary injection of 4 *μ*g of GAD-alum (Diamyd Medical, Stockholm) into an inguinal lymph node, followed by two booster injections of 4 *μ*g each with one-month interval [24, 25]. In parallel, they got vitamin D 2000 U/d per os. Another group of recent-onset T1D patients (*n* = 6, <4 months from diagnosis) participated in a double-blind, placebo-controlled pilot trial (NCT01785108, clinicaltrials.gov) (unpublished), where they received two subcutaneous injections (SC) of GAD-alum, 20 *μ*g each, one month apart (Figure S1). They also in parallel got vitamin D 2000 U/d per os. These patients were selected under blind conditions, before code break according to availability of samples (Figure 1).

### 2.2. Laboratory Tests

Laboratory analyses were performed at Linköping University, Sweden. Blood and serum samples were collected at baseline and after 30, 60, 90, and 180 days in the LN group and after 15, 45, 90, and 180 days in the SC group. Samples were drawn during the morning hours, and PBMCs were isolated within 24 h using Leucosep (Greiner Bio-One) according to the manufacturer's instructions.

### 2.3. Serum Antibodies and IgG Subclasses

Serum GAD autoantibodies (GADA) and IA-2A were estimated in duplicate by means of a radio-binding assay, using ^35^S-labeled recombinant human GAD_65_ as previously described [26]. Sepharose protein A was used to separate free from antibody-bound labeled GAD_65_. A diabetes autoantibody standardization program (IASP) in which the laboratory participated in has shown that the GADA assay has a sensitivity of 70% and specificity of 100% and for IA-2A, 99% sensitivity and 100% specificity.

GADA IgG1, 2, 3, and 4 subclasses were measured by radio-binding assays [27] using IgG subclass-specific biotin-labeled mouse anti-human monoclonal antibodies bound on Streptavidin Sepharose High Performance beads (GE Healthcare Life Sciences, Freiburg, Germany). Mouse anti-human IgG1 (clone G17-1; BD Biosciences, Heidelberg, Germany), IgG2 (clone G18-21; BD Biosciences), IgG3 (clone HP6047; Invitrogen, Carlsbad, CA), and IgG4 (clone JDC-14; BD Biosciences) monoclonal antibodies were used. Nonspecific binding was determined for each serum using biotin-labeled mouse anti-rat IgM monoclonal antibody (clone G53-238; BD Biosciences) bound on Streptavidin Sepharose beads. Briefly, 2 *μ*l of serum was incubated in duplicate with 25,000 cpm of ^35^S-labeled recombinant human GAD_65_ in 25 *μ*l of 50 mM Tris, 150 mM NaCl, and 1% Tween 20 (pH 7.4) (TBST) overnight at 4°C, before addition of 50 *μ*l antibody-coated Streptavidin Sepharose bead suspension, incubation for 1 h at 4°C on a shaker, washing in cold TBST, and counting. Results were expressed as delta cpm (IgG subclass-specific cpm − anti-rat IgM cpm) and converted to arbitrary units (AU) proportional to the GADA IgG subclass-specific delta cpm of a local standard serum.

Total serum IgE was quantified using the ImmunoCap100Є system (Phadia AB, Uppsala, Sweden). The measuring range for the assay was 2–50,000 kU/l, and calibrators were run in duplicate to obtain a full calibration curve. Levels of total IgE ≥ 85 kU/l were regarded as positive.

### 2.4. Lymphocyte Proliferation Assay

PBMCs were incubated in triplicate in round-bottom plates with 5 *μ*g/ml rhGAD65 (Diamyd Medical, Stockholm, Sweden), CD3/CD28 beads (Gibco, Life Technologies AS, Oslo, Norway), and in the medium alone. After 3 days, cells were pulsed for 18 h with 0.2 *μ*Ci of [^3^H] thymidine/well (Perkin Elmer) and harvested thereafter. Proliferation was recorded [28] and expressed as stimulation index (SI) and calculated as the mean of triplicates in the presence of stimulus divided by the mean of triplicates with the medium alone.

### 2.5. Cytokine Secretion Assay

For cytokine quantification, peripheral blood mononuclear cells (PBMCs) were cultured for 7 days in the presence of 5 *μ*g/ml recombinant human GAD65 (Diamyd Medical, Stockholm, Sweden) or in the medium (AIM-V) alone at 37°C in 5% CO_2_, as previously described [11, 28, 29]. The cytokines interleukin- (IL-) 2, IL-5, IL-10, IL-13, IL-17, tumor necrosis factor- (TNF-) *α*, and interferon- (IFN-) *γ* were measured in cell culture supernatants using Bio-Plex Pro Cytokine Panel (Bio-Rad, Hercules, CA, USA) according to the manufacturer's instructions. Data was collected using the Luminex 200™ (Luminex xMAP™ Corporation, Austin, TX, USA). The antigen-induced cytokine secretion level was calculated by subtracting the spontaneous secretion (i.e., secretion from PBMC cultured in the medium alone) from the one following stimulation with GAD65.

### 2.6. Flow Cytometry

PBMCs were incubated in the AIM-V medium with *β*-mercaptoethanol at 37°C, 5% CO_2_ for 7 days, with or without 5 *μ*g/ml recombinant GAD_65_. Thereafter, cells were washed in PBS containing 0.1% BSA and subsequently stained with Alexa 700-conjugated anti-CD3 (clone UCHT1, BD Biosciences), Pacific Blue-conjugated anti-CD4 (clone RPA-T4, BD Biosciences), allophycocyanin- (APC-) H7-conjugated anti-CD8 (clone SK1, BD Biosciences), PerCP-Cy5.5-conjugated anti-CD45RA (clone HI100, BD Biosciences), phycoerythrin- (PE-) conjugated anti-CCR7 (clone G043H7, BioLegend), FITC-conjugated anti-CD127 (clone eBioRDR5, eBioscience), and PE-Cy7-conjugated anti-CD25 (clone BC96, eBioscience). Then, cells were fixed and permeabilized using the FOXP3-staining buffer set (eBioscience), according to the manufacturer's instructions. Cells were then stained with APC-conjugated anti-FOXP3 (clone PCH101, eBioscience) and acquired on a FACS Aria III (Becton Dickinson) running FACS Diva v8 software (Becton Dickinson). Data were analyzed using Kaluza v1.3 (Beckman Coulter).

### 2.7. Ethics

All patients and children's parents gave their oral and written informed consent. Both trials have been approved by the Research Ethics Committee, Linköping University, Sweden (DIAGNODE: Dnr 2014/153-31; DIABGAD-1: Dnr 2012/417-32), and by the Medical Product Agency, Uppsala, Sweden (DIAGNODE: Dnr 5.1-214-54385; DIABGAD-1: Dnr 2012-003251-11).

### 2.8. Statistical Analysis

Data distribution was tested using the Kolmogorov-Smirnov test. Variables that followed a normal distribution were presented as mean (i.e., age, insulin dose, HbA1c, and C-peptide), and differences within groups were calculated by a Student *t*-test. For nonnormally distributed variables, a nonparametric test was applied (Mann–Whitney test). A Wilcoxon test was used for related samples not normally distributed. Differences between categorical variables were calculated by a chi-square test (*χ* ^2^ test). A probability level of <0.05 was considered statistically significant. Calculations were performed using IBM SPSS Statistics version 23 (IBM SPSS, Armonk, NY, USA), and graphical illustrations were made in GraphPad Prism 5 for Windows (GraphPad Software, La Jolla, CA, USA). Due to the small sample size, the immunological and clinical data of the patients were individually represented.

## 3. Results

Patients were stratified into those who received lymph node (LN) or subcutaneous (SC) GAD-alum injections. Gender distribution was the same in both groups, while mean age was higher in LN patients (22 years) than in the SC group (14 years) (*p* = 0.001). Both groups had similar baseline mean C-peptide (fasting, max. stimulated, and AUC). Pretreatment HbA1c values tended to be higher in LN patients (*p* = 0.054), but there was no difference in the insulin dose/kg body weight, 24 hours between the groups. Baseline GADA and IA-2A autoantibody levels did not differ between the groups (Table S1). Follow-up of the patients showed that fasting and stimulated C-peptide (AUC) remained stable at 180 days in the LN group, while HbA1c levels and insulin dose/kg body weight, 24 hours decreased [24]. Patients in the SC group had a greater loss of stimulated C-peptide, as well as an increase in HbA1c and insulin requirement (Table S1).

### 3.1. GADA Titers and GADA Subclass Analysis

**Figure 2:**
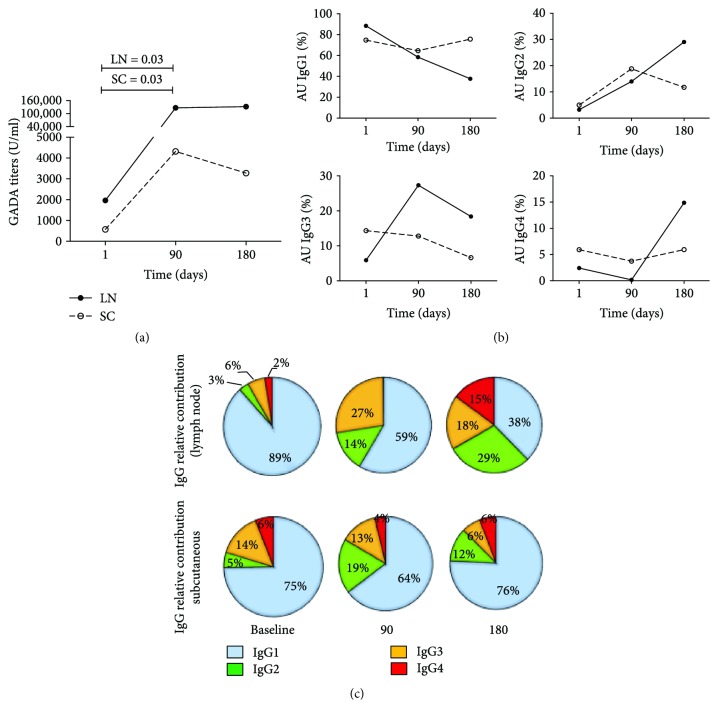
GADA titers and GADA subclass distribution in GAD-alum lymph node and subcutaneous treatment. (a) Median values of GADA titers in the lymph node (LN, *n* = 6) or subcutaneous vaccination (SC, *n* = 6). (b) Change of the frequency (%) of IgG1, IgG2, IgG3, and IgG4 GADA subclasses. Frequencies were calculated with respect to the combined sum of the AUs of the 4 subclasses in each sample. The median percentage with respect to the total IgG is shown for each respective subclass. (c) GADA subclass relative contribution at baseline, 90, and 180 days for LN and SC groups.

GADA levels and GADA subclass distribution at baseline did not differ between the two groups. Following the second injection of GAD-alum given in both SC and LN (Figure 2(a)), GADA levels were enhanced. However, LN administration of low GAD-alum doses induced median GADA 29 times higher than SC injection of higher doses (Figure 2(a)).

We looked next to the longitudinal GADA IgG1–4 subclass responses of the patients, and the distribution of IgG subclasses at each time point was calculated, as frequency of each subclass with respect to the combined sum of the AUs of all the subclasses in each sample. Baseline GADA subclass distribution was similar in the two groups, IgG1 being the most frequent, followed by IgG3 > IgG2 ≈ IgG4 (Figure 2(b), Figure S2). The proportion of IgG1 decreased from baseline to 90 days both in the LN and SC groups, while the proportion of the other subclasses increased. While GADA subclass distribution in the SC group at 180 days was similar to that observed at baseline, the proportion of IgG1 in the LN group was further reduced, with a marked increase of IgG2, IgG3, and IgG4 (Figure 2(c)).

To disregard a possible allergy-associated effect in response of GAD-alum, total IgE was measured at baseline and at 180 days. Baseline IgE levels were similar in patients receiving intralymphatic and subcutaneous injections and were not affected by the treatment but remained unchanged after 180 days (data not shown).

### 3.2. Cytokine Secretion and Relative Contribution

**Figure 3:**
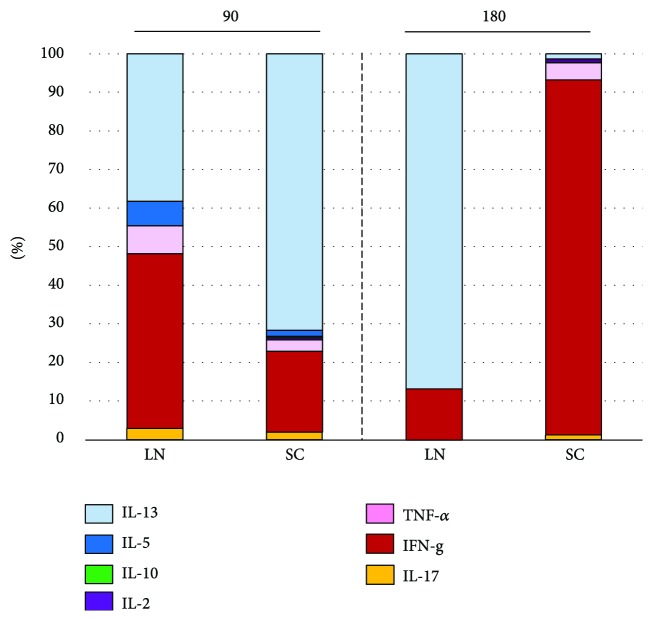
GAD65-induced cytokine secretion upon in vitro PMBC stimulation. Relative contribution (%) of the cytokines in LN patients and SC group at 90 and 180 days.

Baseline cytokine secretion in PBMC supernatants collected after 7 days culture was similar in the two groups. GAD_65_-induced secretion of IL-5, IL-13, IFN-*γ*, and IL-17 was increased at 90 days, both after the second GAD-alum SC and LN doses, together with IL-2 in the SC group. The third GAD-alum injection into the LN resulted in a predominant secretion of IL-13 and low levels of IFN-*γ* at 180 days. Meanwhile, IFN-*γ* was the most secreted cytokine in the SC group at the same time point (Figure S3). We further assessed the relative contribution of each cytokine to the total GAD_65_-induced cytokine secretion. In the LN group, a broad cytokine profile was observed at 90 days, following the second injection of GAD-alum, while cytokine secretion at 180 days, after the third injection, was dominated by the Th2-associated cytokine IL-13. In the SC group, cytokine profile was also characterized by a broad cytokine secretion at 90 days with a predominant secretion of Th2 cytokines, but cytokine distribution shifted in a dominant Th1-like response at 180 days (Figure 3).

### 3.3. In Vitro Stimulation with GAD65

GAD_65_-induced proliferation was increased by the second injection of GAD-alum both into the LN and SC. The third injection to the LN group resulted in a reduction of proliferation at 180 days, while it remained stable in the SC group. Proliferation induced by stimulation with CD3/CD28 beads showed the same distribution as that induced by GAD_65_ (Figure S4).

### 3.4. T Cell Immunophenotype

**Figure 4:**
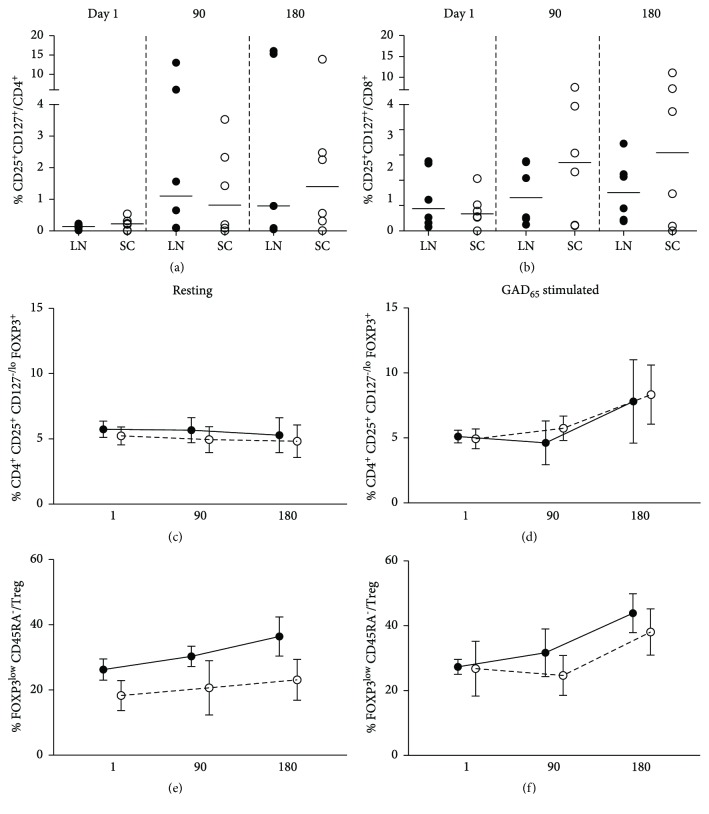
T cell activation induced by GAD65 in lymph node (LN, *n* = 6, black circles) and subcutaneous (SC, *n* = 6, white circles) GAD-alum patients. (a) Percentage of GAD65-activated CD4^+^CD25^+^CD127^+^ T cells and (b) CD8^+^CD25^+^CD127^+^ T cells. (c) Mean percentage of CD4^+^CD25^+^CD127^lo/−^FOXP3^+^ (Treg) in resting samples (medium alone) and (d) induced by GAD65 stimulation. (e) Mean percentage of FOXP3^lo^CD45RA^−^ nonsuppressor regulatory T cells in resting samples (medium alone) and (f) GAD65-stimulated samples.

We monitored the differentiation state and GAD_65_-induced activation of T cells. A representative illustration of the gating strategy for the analysis of CD8, CD4, and regulatory T cells is shown (Figure S5). GAD_65_ stimulation induced activated CD25^+^CD127^+^ T cells in both groups after the second injection of GAD-alum. In the LN, higher frequency of activated CD4 T cells was detected in 3 patients, while activation of CD8 T cells was moderate or not detectable. In the SC group in contrast, the proportion of activated CD8 T cells was more predominant, with weaker expression of GAD_65_ activation of CD4 cells (Figures 4(a) and 4(b)).

The analysis of CD4^+^FOXP3^+^CD25^hi^CD127^low/−^ Tregs showed that resting Treg did not vary through the study, but antigen recall induced cells with regulatory phenotype at 180 days in both groups (Figures S6C and S6D). Further, addition of CD45RA revealed an increment in nonsuppressive FOXP3^lo^CD45RA^−^ T cells in both groups (Figures 4(e) and 4(f)).

CD4 and CD8 T cells were further classified according to the expression of CD45RA and CCR7 as näive (T_N_, CD45RA^+^CCR7^+^), central memory (T_CM_, CD45RA^−^CCR7^+^), effector memory (T_EM_, CD45RA^−^CCR7^−^), and terminally differentiated effector memory (T_EMRA_, CD45RA^+^CCR7^−^) cells (Figure S6). Both groups showed a progressive reduction in the proportion of näive CD4 and CD8 T cells after 90 days, while the frequency of memory and effector cells increased in GAD65-stimulated PBMC (Figure S6).

### 3.5. Identifying Biomarkers of Clinical Outcome

**Figure 5:**
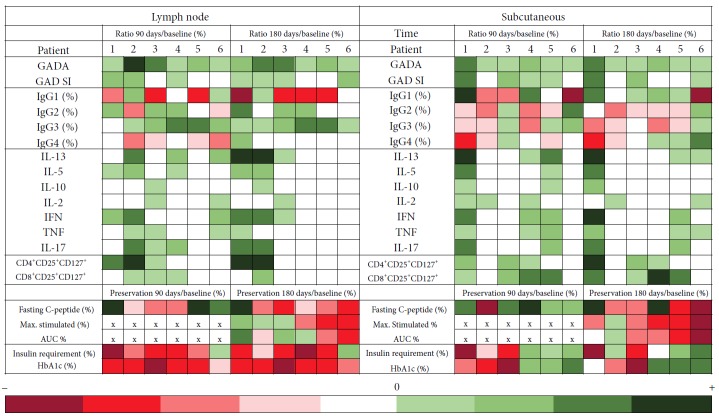
Heat map representing the immunological changes induced by GAD-alum treatment. Variations were calculated as the ratio of the values at 90 and 180 days with respect to the baseline. Patients received GAD-alum injections into the lymph nodes (LNs, *n* = 6) or subcutaneously (SC, *n* = 6), and they were stratified from left to right according to their clinical outcome at 180 days. Clinical variables are expressed as percentage of change from baseline (%). At 90 days, max. stimulated, and AUC C-peptide were not calculated, as meal tolerance tests were not performed, and are represented by “×”. The color scales illustrate the posttreatment increase (green) or decrease (red) of variables in relation to baseline values.

Individual immunological changes induced by GAD-alum treatment were calculated as the ratio of GAD_65_-induced immune responses at 90 and 180 days with respect to immune response pretreatment, and patients were stratified according to their metabolic and C-peptide preservation (Figure 5, Table S2).

Baseline immune response did not show any pretreatment feature that seemed to be related to the clinical outcome. Representation of induced changes of GAD_65_-induced cytokine secretion posttreatment showed that Th1- and Th2-associated cytokines were enhanced after 90 days in most patients, independently of the administration route. However, changes in GAD_65_-induced cytokines at 180 days were detectable in the LN patients with the best clinical response (patients 1, 2, and 3), with lower HbA1c, decreased insulin requirement, and best preservation of C-peptide secretion. Activated CD4 cells but no CD8 were observed in these three patients. Reduction of the proportion of IgG1 and enhancement of IgG2 and IgG4 were most pronounced in the patient with the best clinical response (patient 1), and this patient accounted for the observed increase of IgG4 in the LN group (Figure 5).

GAD_65_-induced cytokine secretion in the patient with best clinical response in the SC group (patient 1) resembled that observed in the LN patients with best response, but in the SC-treated patient, IgG1 was the predominant GADA subclass, and both activated CD4 and CD8 cells were detected.

Calculation of the ratio of Th2 (IL-13 and IL-5)/Th1 (IFN-*γ* and TNF-*α*) cytokines at 180 days for the best responders in both groups (*n* = 2 in LN and *n* = 1 in SC) revealed that Th2 response was three times stronger in LN patients than in the SC group (ratio: 6.44 LN versus 2.24 SC).

## 4. Discussion

In this study, we assessed the influence of the administration route on the immunogenicity induced by GAD-alum treatment. Our results show that direct intralymphatic administration enhanced the immunogenicity of the treatment as compared with subcutaneous delivery. The intensity of the specific immune response induced by low doses of GAD-alum injected into the lymph nodes was comparable with that induced by higher doses given subcutaneously. No specific immune signatures before treatment were identified among the biomarkers included in this study. Although the patients in the SC group were slightly younger, the immunological profile at baseline was quite similar in both groups, supporting the idea that differences in the immune response observed at follow-up were due to the treatment. A common feature for both groups was that patients with better preservation of C-peptide secretion and improved metabolic outcome, that is, lower HbA1c and insulin needs, seemed to have some common immune correlates.

Modifications following therapy included a stepwise increment of GADA in the LN group, with titers peaking after the third injection. Boost of GADA was stronger following low doses given into the LN than by SC administration of larger doses. Intriguingly, GADA increase was associated with the reduction of GAD_65_-induced proliferation after 180 days in the LN patients but not in the SC group. Increased antibody titers and lower proliferation against insulin have also been shown in a prevention trial using intranasal insulin given to at-risk individuals, suggesting induction of tolerance [30]. Reduction of proliferation in the LN group raises the question whether induction of tolerance was part of the immunological effect, but no Treg-associated cytokines or T cells with regulatory phenotype were detected. Instead, GAD_65_-specific CD4 T cell responses were observed. The second injection, both into the LN and SC, induced a cytokine secretion profile dominated by Th2 cytokines. The third LN dose led to a cytokine distribution dominated by the Th2-associated cytokine IL-13 after 6 months, while TNF and IFN were the predominant cytokines in the SC group at the same time point. This is of particular interest, as a cytoprotective action of IL-13 has been shown in pancreatic *β*-cell viability [31], and IL-13 has been described as the main effector of suppression, reducing both Teff proliferation and IL-2 secretion [32]. IFN-*γ* was also detected in the same supernatants, and it could be argued that secretion of Th1-associated cytokines might not be desirable as part of GAD-alum-induced immune responses. However, cytokines can exert different effects depending on their concentrations and microenvironment [33], and IFN-*γ* has a wide-ranging effect on the innate and the adaptive immune system [34]. In that sense, the GAD-alum effect might not only be dependent on the induction of a predominant Th2 response but is also reliant upon a broader range of cytokines that may play a role in restoring the immune balance. T cells with regulatory phenotype were not part of the detected subpopulations, in agreement with results from previous studies [29, 35]. However, as the GAD-alum effect on the immune response is antigen specific [29, 36, 37], it cannot be excluded that the scarce number of GAD_65_-specific cells precluded their identification.

As subclass frequencies can be associated with Th1/Th2 responses, we analyzed whether GAD-alum influenced GADA IgG subclass distribution. A relative reduction of IgG1, with a shift towards IgG2, IgG3, and IgG4 subclasses, was observed in the LN group. It was particularly interesting to observe a high increase of IgG2 that has been described to correlate with IgG4 responses to tetanus vaccine in autoantibody negative children, and both subclasses correlated with IL-4 and IL-13 secretion [38]. The fact that both subclasses were not detectable or were very low in autoantibody-positive children in the same study was interpreted as a reduced capacity to mount Th2-associated responses in prediabetic children. The effect of SC administration of GAD-alum observed in the few patients included in this study is in line with the results from previous GAD-alum trials, where we observed only a transient increase of IgG3 and IgG4 and a reduction of IgG1 after SC administration of 2 doses of the same concentration [26, 28].

Based on the general consensus that T1D is due to the lack of tolerance and the involvement of autoreactive T cells, it has been expected that efficacy of immunotherapy with autoantigens should be accompanied by the induction of tolerance. Here, we observe that immune responses in individuals with better clinical outcome after LN injections were characterized by rise in the GADA levels, lower proliferation, and predominant Th2-like responses, supported by increase in IL-13 and shift of IgG subclasses. Although the direction of the immune responses to GAD_65_ was Th2 skewed in the LN group, there were however large interindividual differences. Strikingly, GAD_65_-induced cytokines were detected in the LN group after 180 days solely in patients with the best clinical outcome. It could be argued that lack of T cell responses might be due to failure of injections into the lymph node. However, the GADA enhancement in those patients suggests that this explanation is less likely. It was particularly interesting that patients displaying a better clinical outcome were characterized by GAD-induced T cell responses deviated towards a Th2 cytokine profile, both after SC and LN treatments. We have shown that SC injection of 2 doses of GAD-alum induced a broad cytokine secretion that switched towards a more predominant Th2-associated profile over time [28]. Administration of further SC doses in the phase III study increased the secretion levels but did not affect the quality of the cytokine response, and cytokine profile was similar in patients receiving 2 or 4 doses of GAD-alum [28]. Thus, a different cytokine secretion profile after the third LN injection cannot be explained by an extra dose but rather by the administration route. Intralymphatic administration delivers more antigen to the site of immune response induction [39], and the difference in antigen dose available for stimulation of antigen-specific T cells may also lead to increased Th2 response. The adjuvant aluminum effect is associated with the induction of Th2 responses [40] and preferentially induces humoral rather than cellular immune responses. Therefore, alum has been the adjuvant of choice to minimize the possibility of promoting *β*-cell-mediated destruction together with GAD_65_. We could not identify any pretreatment signature for patients who responded better. However, each individual presented unique clinical and immunological features, and it is possible that modest differences or other parameters not included in this study can have an impact on treatment outcome.

One of the main problems in the development of T1D treatment has been the extrapolation of results from animal models to humans, proven to be more difficult and less accurate than expected. Indeed, besides patient heterogeneity, the possible interference of many other factors as infections or medication during treatment might be more relevant than what has been considered when looking at the effect at group levels. In this open trial, we had the possibility to dissect the events along treatment and made some interesting observations. For instance, one of the patients in the LN group seemed to get a specific response to GAD_65_ after 90 days but that faded at the later control. That patient reported recurrent colds after the 90-day period and antihistaminic and health supplement intake, as well as the use of corticosteroid cream. Two other patients without detectable cytokine secretion were taking antihistaminic pills, and one of them even antidepressive medication. No concomitant medication of any type was reported for the patient responding clinically best and who displayed the strongest treatment-associated immunological changes. We have recently shown that H1N1 vaccination might have had an impact on the outcome of the phase III trial and that signs of possible vaccine interference were observed far beyond the time period during which concomitant vaccinations, excluding influenza, were forbidden in the trial [11]. Thus perhaps, it is reasonable to consider whether failure of reaching differences in placebo-controlled trials must immediately lead to treatments disregard. A beneficial effect in some patients might be good enough to support the use of the agent alone or in combination with other agents, in efforts to tailor treatment for each patient. Our results highlight the relevance of this kind of small pilot trials in treatment development, as they can give faster insight in many unsolved questions and help to speed experimental treatment modalities towards successful clinical use.

## 5. Conclusion

Intralymphatic administration of autoantigen in T1D might represent a promising therapeutic approach to increase the efficacy of autoantigen immunotherapy, although our observations are based on a small number of patients and have to be interpreted cautiously. Whether the immunological changes associated with clinical efficacy observed in the participants in this pilot trial might be regarded as a favorable immune outcome must be addressed in future larger double-blind studies. Nevertheless, results from small pilot trials are needed to test new approaches before the initiation of full-scale, randomized, controlled trials.

## Data Availability

The data used to support the findings of this study are available from the corresponding author upon request due to patient privacy.

## Supplementary Materials

Supplementary Materials Table S1: baseline clinical and immunological parameters in the patients who received GAD-alum injections into the lymph nodes (LNs, *n* = 6) or subcutaneously (SC, *n* = 6). Table S2: clinical and immunological parameters at 90 and 180 days in the patients who received GAD-alum injections into the lymph nodes (LNs, *n* = 6) or subcutaneously (SC, *n* = 6). Figure S1: protocol of the GAD-alum treatment. Figure S2: GADA IgG1–4 subclasses expressed as arbitrary units (AU) in follow-up serum samples. (A) Lymph node patients and (B) subcutaneous vaccination. Figure S3: median levels of IL-13, IL-5, IL-10, IL-2, IL-17, TNF-*α*, and IFN-*γ* (pg/ml) at baseline, 90, and 180 days in lymph node (LN, black circles, *n* = 6) and subcutaneous (SC, white circles, *n* = 6) GAD-alum patients. Figure S4: median values of proliferative responses to GAD65 and CD3/CD28 beads in GAD-alum patients at baseline, 90, and 180 days. Figure S5: flow cytometry gating strategy. Figure S6: change of differentiation state (%) of CD4^+^ and CD8^+^ T cells in lymph node (LN, *n* = 6, black circles) and subcutaneous (SC, *n* = 6, white circles) GAD-alum patients.
