# Supplementary material for: Intralymphatic Glutamic Acid Decarboxylase-Alum Administration Induced Th2-Like-Specific Immunomodulation in Responder Patients: A Pilot Clinical Trial in Type 1 Diabetes
Source: J Diabetes Res. 2018 May 24;2018:9391845. doi: 10.1155/2018/9391845 (PMC5994289; doi:10.1155/2018/9391845)
Supplement: Supplementary Materials — Table S1: baseline clinical and immunological parameters in the patients who received GAD-alum injections into the lymph nodes (LNs, n = 6) or subcutaneously (SC, n = 6). Table S2: clinical and immunological parameters at 90 and 180 days in the patients who received GAD-alum injections into the lymph nodes (LNs, n = 6) or subcutaneously (SC, n = 6). Figure S1: protocol of the GAD-alum treatment. Figure S2: GADA IgG1–4 subclasses expressed as arbitrary units (AU) in follow-up serum samples. (A) Lymph node patients and (B) subcutaneous vaccination. Figure S3: median levels of IL-13, IL-5, IL-10, IL-2, IL-17, TNF-α, and IFN-γ (pg/ml) at baseline, 90, and 180 days in lymph node (LN, black circles, n = 6) and subcutaneous (SC, white circles, n = 6) GAD-alum patients. Figure S4: median values of proliferative responses to GAD65 and CD3/CD28 beads in GAD-alum patients at baseline, 90, and 180 days. Figure S5: flow cytometry gating strategy. Figure S6: change of differentiation state (%) of CD4+ and CD8+ T cells in lymph node (LN, n = 6, black circles) and subcutaneous (SC, n = 6, white circles) GAD-alum patients. [file 9391845.f1.pdf]

Supplementary material

Table S1. Baseline clinical and immunological parameters in the patients who received GAD-alum injections into the lymph-nodes (LN, n=6) or subcutaneously (SC, n=6).

Baseline													
Variables	Lymph-node (n=6)						Subcutaneous (n=6)						P value
Age (years)	23	22	21	21	23	21	13	17	17	11	15	11	<0.001
Gender	M	M	M	F	M	F	M	F	M	F	M	M	0.990
Fasting C-peptide (nmol/L)	0,12	0,26	0,26	0,25	0,16	0,17	0,18	0,59	0,24	0,15	0,48	0,18	0,237
Max.stimulated	0,61	1,13	0,62	1,26	0,73	0,58	0,99	0,9	0,77	0,56	1,12	0,61	0,983
AUC	0,43	0,85	0,42	0,74	0,38	0,36	0,69	0,8	0,6	0,46	0,97	0,44	0,302
Insulin requirement (IU/kg/24hr)	0,29	0,25	0,29	0,45	0,55	0,46	0,55	1,36	0,6	0,38	0,42	0,48	0,143
HbA1c (nmol/mol)	66	58	103	68	78	52	47	68	65	50	33	41	0,054
GADA (U/ml)	929	111	968	2955	14100	27450	114	786	667	70	472	1215	0,140
IgG1 (%)	70	25	64	91	92	43	39	64	87	10	87	85	0,900
IgG2(%)	29	55	9	0,3	0,2	17	8	6	1,4	59	1,7	4,7	0,701
IgG3(%)	0,4	5,8	11,5	8,7	5,6	17,9	16	19	10,4	28	9	3,6	0,190
IgG4(%)	0,9	14,5	14,7	0,3	2,7	21	37	10,2	1,4	3	2,6	7	0,861
IA-2 (U/ml)	704	266	497	13,6	2,7	2,7	23300		2880	619		4,7	0,181
IL-13 (pg/ml)	nd	nd	nd	nd	nd	nd	nd	nd	969	nd	nd	nd	_
IL-5 (pg/ml)	8.6	0.6	nd	nd	nd	0.6	nd	nd	80.5	nd	nd	nd	_
IL-10 (pg/ml)	0.95	nd	nd	nd	nd	nd	0.25	nd	3.74	1.35	nd	nd	_
IL-2 (pg/ml)	nd	nd	nd	0.61	nd	nd	nd	nd	nd	nd	nd	2.36	_
IFN-g (pg/ml)	nd	nd	nd	nd	nd	nd	nd	nd	1976.03	nd	3.51	nd	_
TNF-a (pg/ml)	nd	nd	nd	nd	nd	nd	nd	nd	37.1	0.78	nd	nd	_
IL-17 (pg/ml)	nd	nd	nd	nd	nd	nd	nd	nd	10.2	1.3	nd	nd	_
Change basal-180 days Fasting C-peptide	108,33	-23,07	-26,92	-4	-25	-47,05	77,77	-18,65	-20,83	153,33	-41,66	-94,44	0,784
Change basal-180 days Max.Stimulated	13,11	7,96	3,22	-11,11	-31,5	-31,03	-22,22	7,77	-15,58	-28,57	-32,14	-60,65	0,192
Change basal-180 days AUC	30,23	-1,17	21,42	6,75	-13,15	-27,77	-5,79	6,25	-11,66	-19,56	-35,05	-65,9	0,101
Change basal-180 days Insulin requirement	-48,27	-4	-41,37	-64,44	-27,27	17,39	-54,54	9,55	-46,66	65,78	16,66	41,66	0,178
Change basal-180 days HbA1c	-31,81	-32,75	-55,33	-47,05	-44,87	-15,38	2,12	-19,11	-55,38	48	36,36	39,02	0,024

C-peptide (nmol/L), HbA1c (nmol/mol) levels, insulin intake (IU/kg/24hr), pre-treatment GADA and IA-2 titers (U/ml), GADA IgG subclass relative distribution (%), and levels of GAD65-induced cytokine secretion (pg/ml) from each patient. M: male, F: female. N.D : no detectable levels. Percentage of change (%) of C-peptide, insulin intake and HbA1c from baseline to 180 days (Δ variable change) were calculated and displayed in the right side of the table.

Table S2. Clinical and immunological parameters at 90 and 180 days in the patients who received GAD-alum injections into the lymph-nodes (LN, n=6) or subcutaneously (SC, n=6).

	Id	Fasting C-peptide (nmol/L)	Max. Stim	AUC	Insulin intake (IU/kg/24 hr)	HbA1c (nmol/mol)	GADA (U/ml)	GAD SI	IgG1 (%)	IgG2 (%)	IgG3 (%)	IgG4 (%)	IL-13 pg/ml	IL-5 pg/ml	IL-10 pg/ml	IL-2 pg/ml	IFN pg/ml	TNF pg/ml	IL-17 pg/ml
90 days																			
LN	1	0.29	x	x	0.11	41	1653	4.1	56.74	43.09	0.03	0.11	n.d	22.58	1.5	n.d	44.33	n.d	n.d
	2	0.24	x	x	0.22	40	136800	2.87	48.37	41.88	9.25	0.48	1377.63	209.41	n.d	n.d	476.66	26.09	52.94
	3	0.21	x	x	0.18	43	413300	0.3	25.94	33.32	33.6	7.12	n.d	n.d	0.33	6.54	n.d	3.69	1.73
	4	0.28	x	x	0.23	43	20580	1.12	31.69	19.05	48.97	0.27	84.29	3.54	n.d	n.d	n.d	n.d	11.2
	5	0.31	x	x	0.43	40	333100	0.53	57.16	0.11	42.68	0.03	n.d	n.d	n.d	n.d	n.d	n.d	n.d
	6	0.22	x	x	0.49	48	116400	0.86	49.52	11.21	29.85	9.39	22.77	n.d	n.d	4.46	27.16	9.45	n.d
SC	1	0.24	x	x	0.17	42	40270	14.55	85.48	2.74	9.97	1.80	3553.29	315.45	8.93	2.77	839.16	23.71	60.17
	2	0.28	x	x	1.32	52	4104	n.d	50.74	27.73	12.57	8.93	n.d	n.d	n.d	20.93	n.d	n.d	n.d
	3	0.32	x	x	0.31	32	3374	3.11	72.46	6.92	17.98	2.63	389.02	9.26	1.9	n.d	88.3	6.1	11.85
	4	0.47	x	x	0.47	59	3072	0.12	51.40	37.58	6.96	4.04	75.54	n.d	0.56	9.35	47.27	12.91	1.27
	5	0.54	x	x	0.43	39	4530	0.57	87	0.30	12.38	0.30	500.48	24.82	6.9	n.d	1817.97	92.88	19.29
	6	0.22	x	x	0.58	60	6700	n.d	26.80	43.56	22.57	7.06	n.d	n.d	n.d	1.33	n.d	n.d	n.d
180 days																			
LN	1	0.25	0.69	0.56	0.15	45	20490	1.92	15.48	61.74	4.02	18.76	2360.79	242.26	n.d	n.d	210.06	274.69	135.47
	2	0.2	1.22	0.84	0.24	39	110000	0.54	29.87	48.71	7.84	13.56	8044.46	400.79	8.17	n.d	465.85	n.d	135.66
	3	0.19	0.64	0.51	0.17	46	596000	0.12	31.11	28.51	27.38	12.98	15.88	n.d	n.d	19.49	2.34	n.d	n.d
	4	0.24	1.12	0.79	0.16	36	25600	n.d	43.01	18.04	38.61	0.33	n.d	n.d	n.d	n.d	n.d	n.d	n.d
	5	0.12	0.5	0.33	0.4	43	673000	0.09	63.76	0.13	36.05	0.04	n.d	n.d	n.d	n.d	n.d	n.d	n.d
	6	0.09	0.4	0.26	0.54	44	154600	5.42	40.67	15.27	22.81	21.23	n.d	n.d	n.d	n.d	n.d	n.d	n.d
SC	1	0.32	0.77	0.65	0.25	48	39920	11.67	84.48	7.45	3.33	4.72	9060.62	554.72	28.5	n.d	2080.64	75.29	130.71
	2	0.48	0.97	0.85	1.49	55	2229	n.d	64.49	16.29	12.17	7.03	n.d	n.d	n.d	6.4	n.d	n.d	n.d
	3	0.19	0.65	0.53	0.32	29	2006	4.36	88.48	0.33	10.83	0.33	1557.65	159.52	6.11	11.43	697.88	51.44	13.81
	4	0.38	0.4	0.37	0.63	74	2032	n.d	34.46	53.70	4.03	7.79	n.d	n.d	n.d	n.d	n.d	n.d	n.d
	5	0.28	0.76	0.63	0.49	45	4324	n.d	91.16	0.34	3.78	4.70	5.65	n.d	n.d	n.d	2165.54	30.27	9.35
	6	0.01	0.24	0.15	0.68	57	5550	1.35	31.71	20.93	5.98	41.36	4.4	n.d	n.d	6.13	n.d	3.81	n.d

C-peptide (nmol/L) and HbA1c (nmol/mol) levels, insulin requirement (IU/kg/24hr), GADA titers (U/ml), GADA IgG subclass relative distribution (%), and levels of GAD65-induced cytokine secretion (pg/ml) from each patient. n.d: no detectable levels.

Figure S1. Protocol of the GAD-alum treatment.

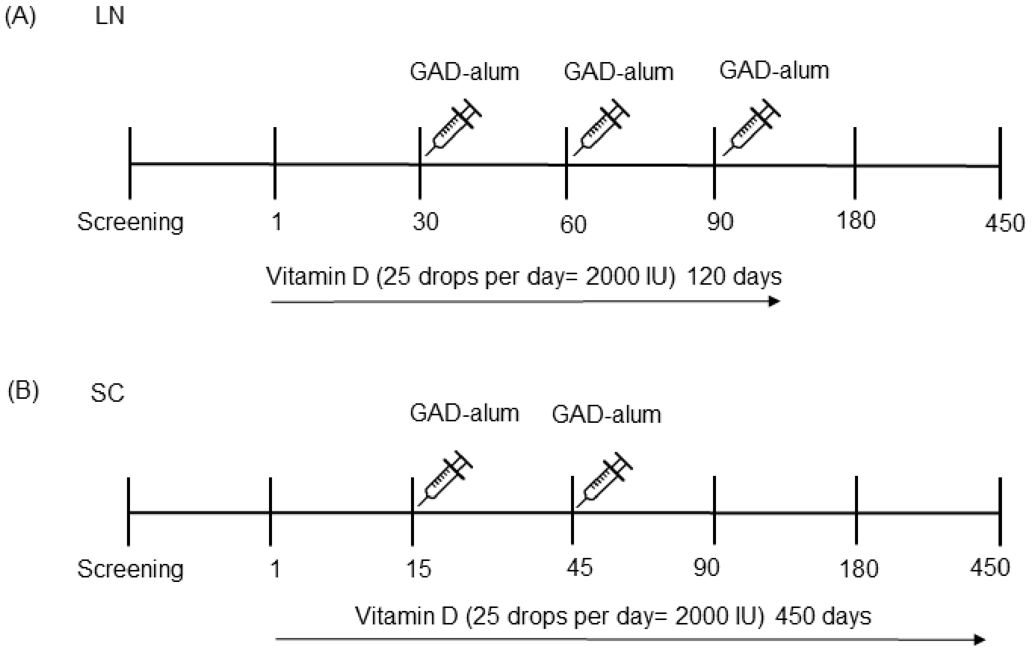

(A) Type 1 diabetic patients (n=6) got a first injection of GAD-alum (4µg) into the lymph-nodes (LN) , followed by two booster injections one month apart. (B) A group of patients who participated in another study (n=6) have received a first subcutaneous (SC) GAD-alum dose (20 µg) followed by a second injection after one month. All patients were also taking Vitamin D (Calciferol) from day 1, during 120 days in the LN patients and for 450 days in the SC group.

Figure S2 . GADA IgG 1-4 subclasses expressed as arbirtrary units (AU) in follow-up serum samples.

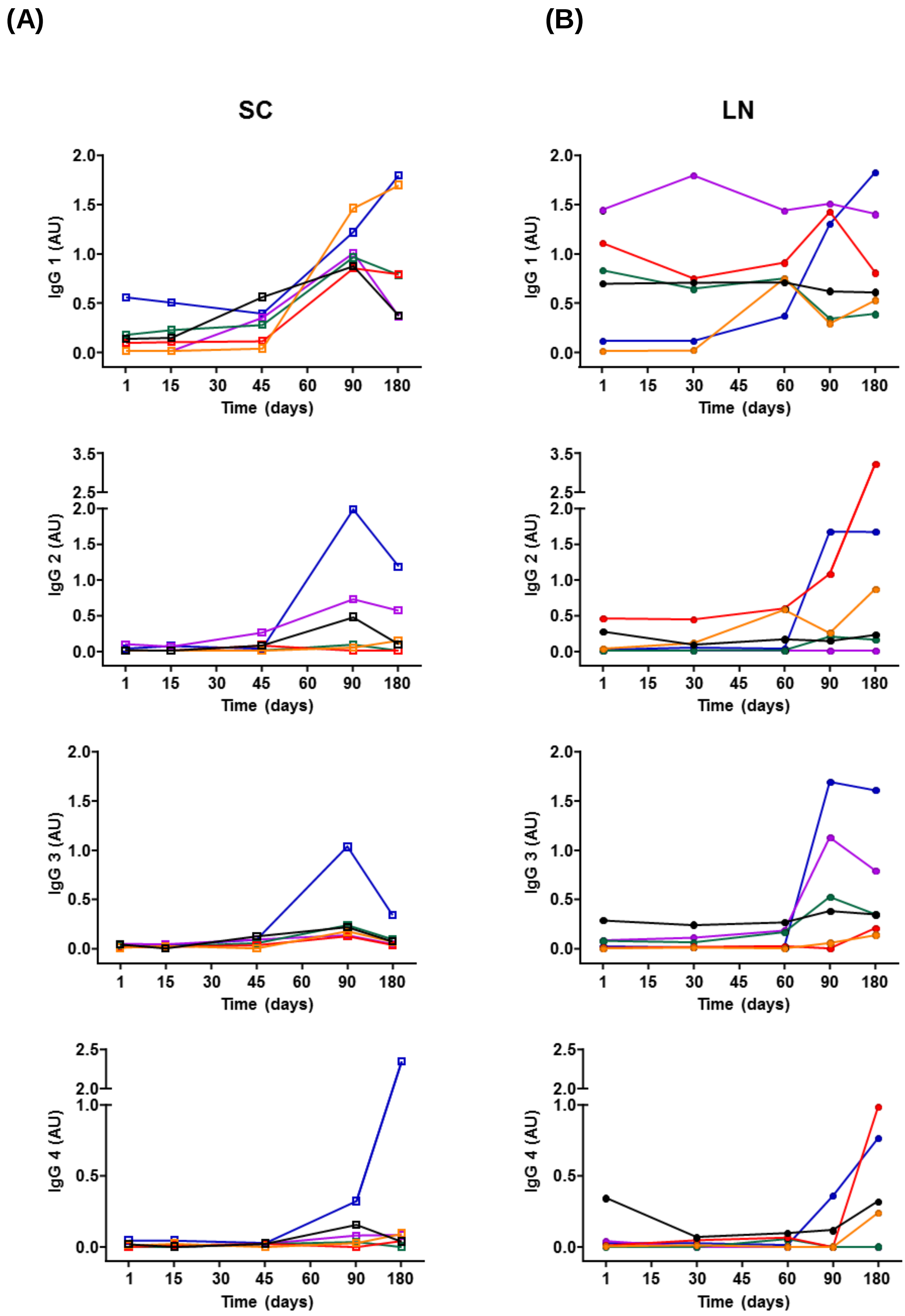

(A) Lymph-node patients and (B) subcutaneous vaccination. Samples of individual patients are connected by lines. For each IgG subclass, results of individual patients are presented using the same colour and symbol.

Figure S3. Median levels of IL-13, IL-5, IL-10, IL-2, IL-17, TNF-α and IFN-γ (pg/ml) at baseline, 90 and 180 days in lymph-node (LN, black circles,n=6) and subcutaneous (SC, white circles, n=6) GAD-alum patients. GAD65-induced cytokine secretion is given after subtraction of spontaneous secretion from each individual.

(A)

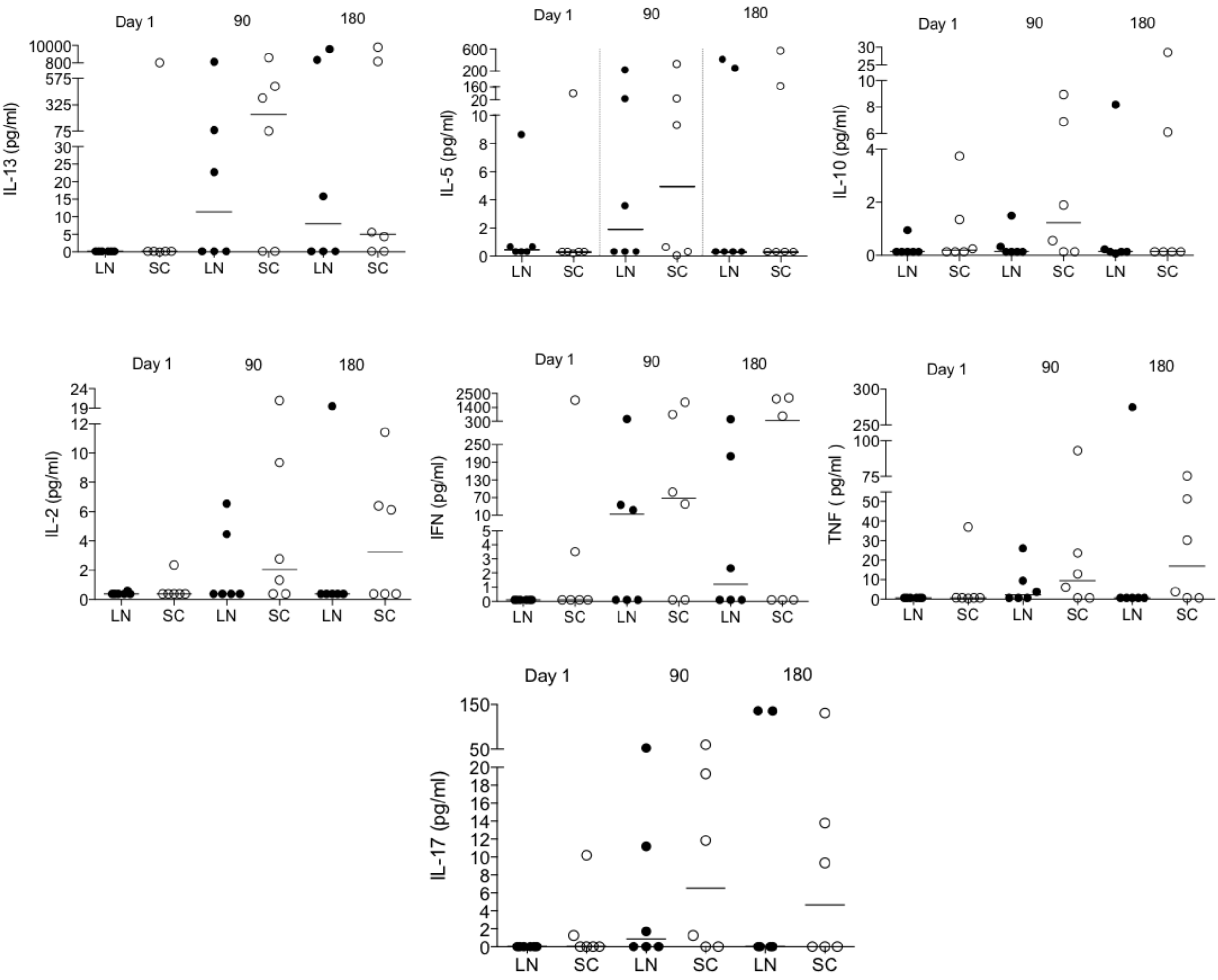

Figure S4. Median values of proliferative responses to GAD65 and CD3/CD28 beads in GAD-alum patients at baseline, 90 and 180 days .

(A)

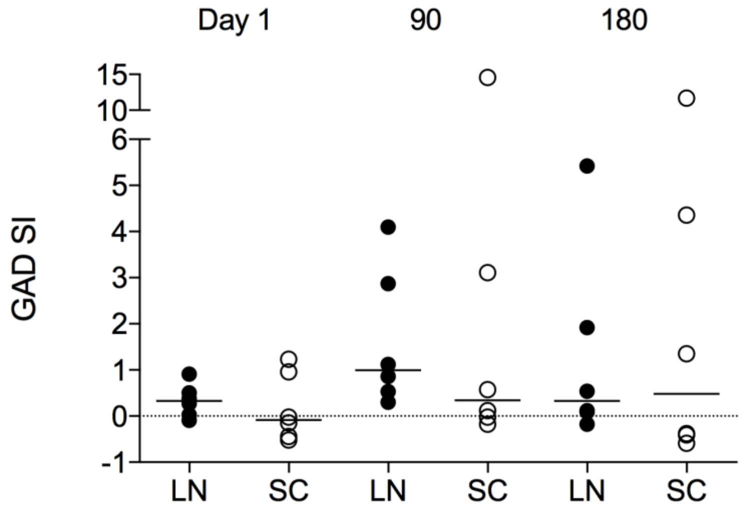

(B)

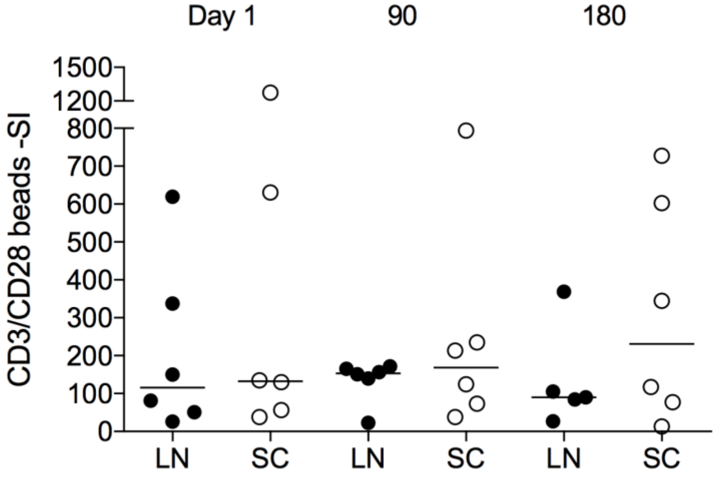

(A) GAD65 (5 µg/ml) and (B) CD3/CD28 beads SI in lymph-node patients (black circles) and subcutaneous group (white circles) Proliferation is expressed as stimulation index (SI), calculated from the mean of triplicates divided by the mean of triplicates with medium alone. SI scale started from 0 after subtracting unstimulated value index (SI unstimulated=1).

Figure S5. Flow cytometry gating strategy.

(A)

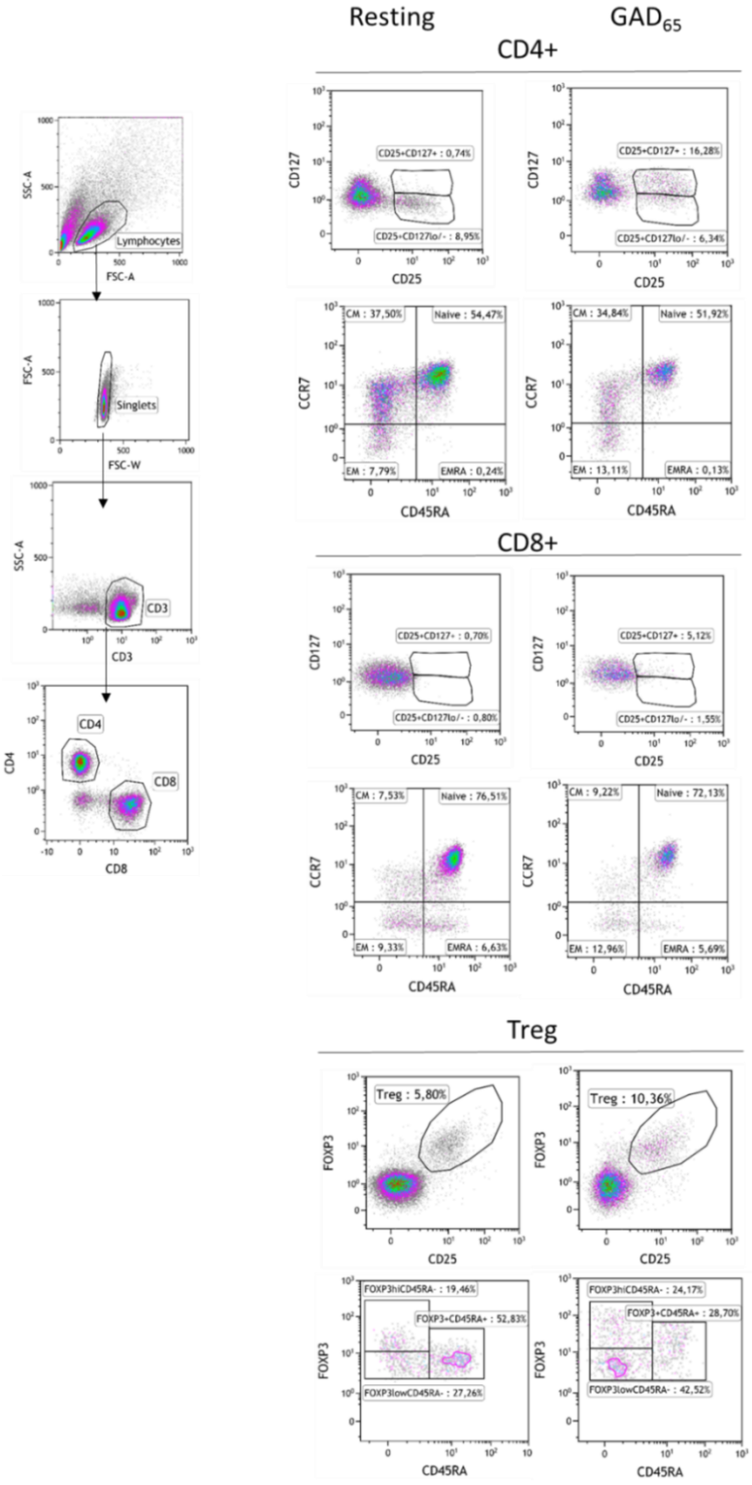

(A) Representative analysis from one patient who received lymph node injections of GAD-alum . The sample correspond the 180 days control. PBMC were cultured for 7 days in the presence of GAD65 (5µg/ml) or medium. The percentages of CD4+, CD8+ T cells and Tregs were assessed in resting (medium alone) and GAD65-stimulated sample.

Figure S6. Change of differentiation state (%) of CD4+ and CD8+ T cells in lymph node (n=6, LN, black circles) and subcutaneous (n=6, SC, white circles) GAD-alum patients.

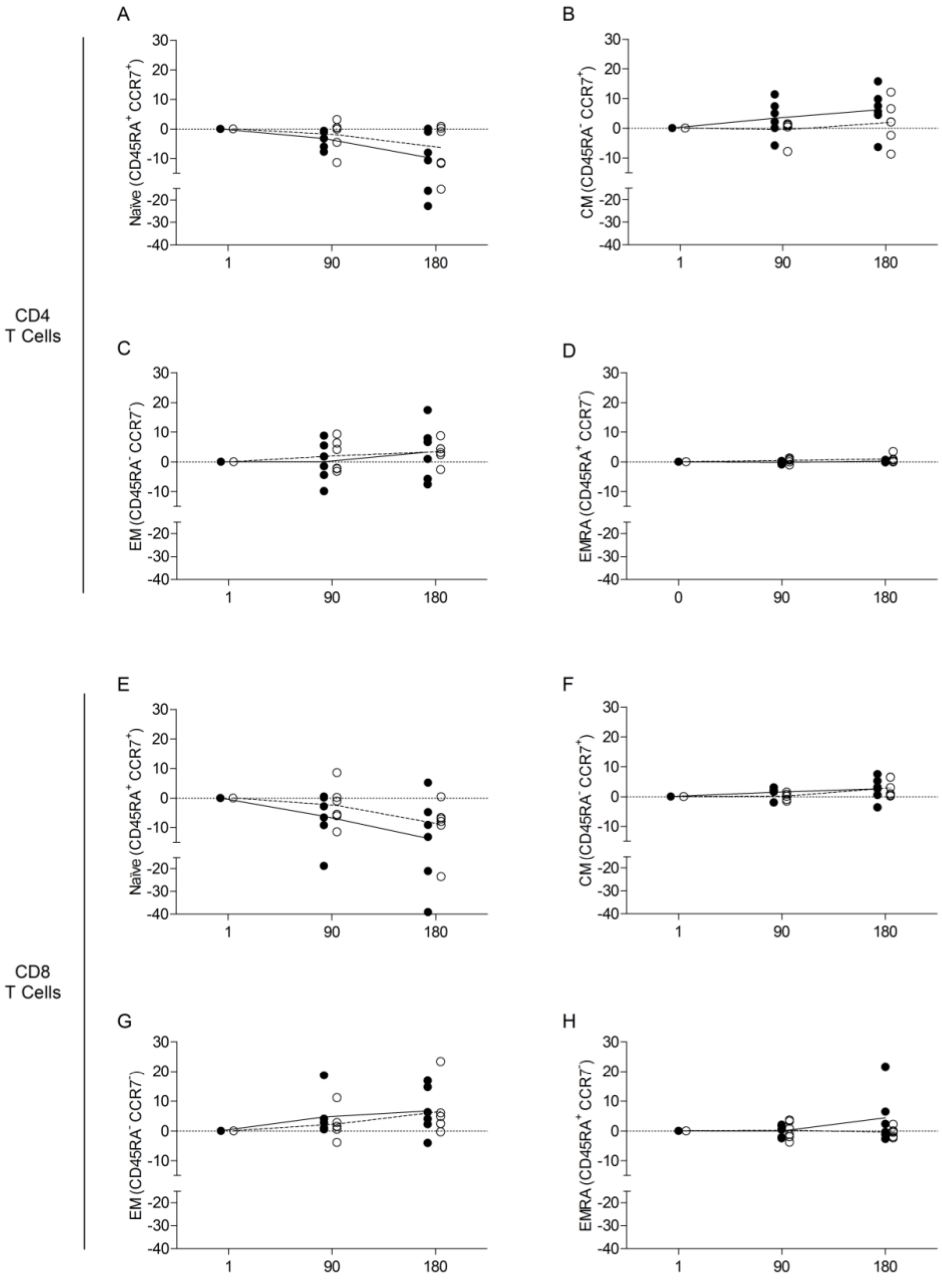

(A) GAD65 induced changes of Näive (CD45RA+CCR7+), (B) central memory (CM, CD45RA-CCR7+), (C) effector memory (EM, CD45RA-CCR7-) and (D) terminally differentiated effector memory (EMRA, CD45RA+CCR7-) CD4 T cells. (E) GAD-alum induced change of näive (CD45RA+CCR7+), (F) central memory (CM, CD45RA-CCR7+) (G) percentage effector memory (EM, CD45RA-CCR7-) and (H) terminally differentiated effector memory (EMRA, CD45RA+CCR7-) CD8 T cells. Lines represent mean trend
